# Melanocytic Nevi as Biomarkers of Breast Cancer Risk

**DOI:** 10.1371/journal.pmed.1001661

**Published:** 2014-06-10

**Authors:** Barbara Fuhrman, Victor Cardenas

**Affiliations:** Department of Epidemiology, Fay W. Boozman College of Public Health, University of Arkansas for Medical Sciences, Little Rock, Arkansas, United States of America

## Abstract

In a linked Perspective, Barbara Fuhrman and Victor Cardenas discuss the potential biological mechanisms that may underlie the association between nevi and risk for breast cancer.

*Please see later in the article for the Editors' Summary*

**Linked Research Articles**

This Perspective discusses the following new studies published in *PLOS Medicine*:

Zhang M, Zhang X, Qureshi AA, Eliassen AH, Hankinson SE, et al. (2014) Association between Cutaneous Nevi and Breast Cancer in the Nurses' Health Study: A Prospective Cohort Study. PLoS Med 11(6): e1001659. doi:10.1371/journal.pmed.1001659

Using data from the Nurses' Health Study, Jiali Han and colleagues examine the association between number of cutaneous nevi and the risk for breast cancer.

Kvaskoff M, Bijon A, Mesrine S, Vilier A, Baglietto L, et al. (2014) Association between Melanocytic Nevi and Risk of Breast Diseases: The French E3N Prospective Cohort. PLoS Med 11(6): e1001660. doi:10.1371/journal.pmed.1001660

Using data from the French E3N prospective cohort, Marina Kvaskoff and colleagues examine the association between number of cutaneous nevi and the risk for breast cancer.

In this week's issue of *PLOS Medicine*, Jiali Han and colleagues [1] and Marina Kvaskoff and colleagues [2] describe prospective studies of melanocytic nevi and breast cancer risk among middle-aged women. Melanocytic nevi, commonly known as moles, are a heterogeneous group of benign tumors of the skin, which are commonly acquired in childhood and adolescence and which may disappear with increasing age [3]. Nevi are a common phenotypic trait and a recognized risk factor for malignant melanoma [4], but have not, to our knowledge, been investigated previously as a risk factor for breast cancer. In these separate, large cohort studies, investigators have observed, unexpectedly, small but significant increases in breast cancer risk across categories reflecting greater numbers of melanocytic nevi.

The analysis conducted by Han and colleagues [1] draws its participants from the Nurses' Health Study (NHS) cohort, comprising 74,523 white women in the United States who were aged 40–65 y in 1986 when they responded to a self-administered questionnaire that asked each respondent to count and report the number of nevi of diameter ≥3 mm on her left arm, inspected from shoulder to wrist. Participants were followed through 2010, accruing more than 1.5 million person-years, and in that time 5,483 cases of invasive breast cancer were ascertained. Following adjustment for known breast cancer risk factors, participants reporting the most nevi (15+) were 35% more likely to be diagnosed with breast cancer than their counterparts who reported no nevi (*p* for trend = 0.003).

The analysis conducted by Kvaskoff and colleagues [2] was carried out using data from the E3N Teachers' Study Cohort, and included 89,902 women in France who were aged 39–66 when they enrolled in the study between 1989–1991. At baseline, participants were asked to report on their number of moles using qualitative categories as follows: “none,” “a few,” “many,” or “very many.” The cohort was followed through 2008; a total of 5,956 incident breast cancer cases were ascertained over nearly 1.4 million person-years. In age-adjusted models, women reporting the most nevi were observed to have 13% higher breast cancer risk than their counterparts without nevi (*p* for trend = 0.04). This association was attenuated and the trend no longer significant when investigators adjusted for benign breast disease or family history of breast cancer. A non-statistically significant increase in breast cancer risk of 8% was observed after adjusting for established breast cancer risk factors and measures of ultraviolet radiation exposure.

While their findings are broadly consistent, subgroup findings in the two studies were different. Kvaskoff and colleagues found no significant association in postmenopausal women, but observed that, among premenopausal women, those who had reported “very many” nevi were 34% more likely to be diagnosed with breast cancer compared to counterparts who reported “none” even after adjustment for all potential confounders (*p* for trend = 0.03, *p* for heterogeneity = 0.04). In contrast, Han and colleagues found no significant modification of the risk association by menopausal status.

The association between nevi and breast cancer risk is unlikely to be causal. Both melanocytic nevi and melanoma are derived from melanin-producing cells in dermal or epidermal tissues [5]; in contrast, most breast cancers are thought to arise from epithelial cells of ductal or lobular origin [6]. Given their distinct origins and locations, nevi are unlikely to act as intermediates or causal agents in the pathway to breast cancer; it seems likely instead that the observed associations reflect a shared cause or causes.

Nevi may be a marker of exposure to sex hormones. Estrogens play established roles in the etiology and pathobiology of breast tumors [7]. Several epidemiologic features of melanocytic nevi are also suggestive of a causal role for sex hormones including an observed peak during puberty in the acquisition and prevalence of nevi [8], and the clinical observation that pregnancy is frequently associated with changes in the appearance and size of moles [9]. Experimental studies have shown that melanocytic nevi have estrogen receptors [10], and melanocytes have been observed to proliferate and to increase melanin production in response to estrogen exposure [11]. Other studies have pointed to associations of melanocytic nevi with other non-malignant proliferative conditions that are also thought to be estrogen-related, including endometriosis [12] and uterine leiyomyoma [13].

The hypothesis that sex steroid hormones are a shared cause of melanocytic nevi and breast cancer was tested directly by Han and colleagues [1]. They first compared, cross-sectionally, plasma estradiol and testosterone concentrations in postmenopausal women by categorical nevi counts and observed that free estradiol and free testosterone rose significantly across categories of greater numbers of nevi. They then carried out a case-control comparison nested among postmenopausal women who could be classified both by nevi counts and by circulating estrogen measures. Han and colleagues observed that adjustment for free estradiol resulted in substantial attenuation of the association between nevi and breast cancer risk, thus supporting the hypothesis.

In addition, nevi counts were observed to be associated with a number of established breast cancer risk factors [1],[2]. In both studies nevi counts were higher in younger women, taller women, women reporting earlier menarche, and women reporting a history of benign breast disease. Nevi were also consistently associated with use of exogenous hormones in pre- and postmenopause. While many of these risk factors are consistent with a causal model that posits exposure to estrogens as a common cause acting across the life course, some observed associations point to other factors; the association of nevi with height, for example, may point to pleiotropic effects of growth hormone or other somatotropic factors on skin and breast development.

The major appeal of these findings comes from the idea, as yet untested, that nevi may be a useful marker of breast cancer risk. Both primary prevention and screening efforts will be more effective if we can better identify and target women who are truly at high risk of the disease. Novel markers of risk may improve the ability of these models to discriminate between women who will and will not go on to develop breast cancer [14]. Counts of melanocytic nevi are non-invasive and easily accomplished in both young and older women and may integrate histories of exposure and susceptibility factors such as host genetics.

Unfortunately, the estimates of association presented in these studies suggest that the independent effect of self-reported nevi count on breast cancer risk is weak and therefore the marker may contribute only modestly to predictive models of individual risk. Observed attenuation of risk estimates following adjustment for several other known risk factors suggests that the marker may be most usefully applied in women who lack other information about their risk of breast cancer, such as women with unknown family histories or those who have not yet undergone an initial mammographic screening. While the finding by Kvaskoff and colleagues, that the association between nevi counts and breast cancer risk was significantly modified by menopausal status, was not replicated in the study by Han and colleagues, it does remind us that there may be population subgroups in which nevi perform better as predictors of risk. It should also be considered that, according to SEER data from 2008–2010, the absolute risks of invasive breast cancer in middle-aged white women (aged 40–65) are approximately 7 times higher than the risk of malignant melanoma [15]. Given this difference, one can expect many more nevi-associated breast cancers than nevi-associated melanoma cases in spite of the substantially higher relative risk of melanoma previously observed in association with nevi counts [4].

Many unresolved questions remain. Will observed associations prove robust across studies and populations? Will the marker prove less informative in non-white populations or in those with less intense or more episodic sun exposure? Some previous studies suggest that research participants tend to undercount nevi and that reliability of nevi counts is limited [16]; therefore, might clinical assessments of nevi be more informative than counts carried out by women themselves? Would refinements to the phenotypic measure be helpful in improving utility of the marker for risk prediction—for example, could atypical nevi be more informative than all nevi with respect to breast cancer risk? Additional studies should be carried out to investigate melanocytic nevi and other cutaneous features in association with the risks of breast cancer and other estrogen-related proliferative diseases. It is our hope that this research will provide etiologic insights and test practical uses of nevi and related phenotypes for their utility in breast cancer risk assessment.
